# Homage to Ebola Fighters: Black Labor and Humanitarian Media Campaigns

**DOI:** 10.1111/maq.12710

**Published:** 2022-05-30

**Authors:** Veronica Gomez‐Temesio

**Affiliations:** ^1^ Global Studies Institute University of Geneva

## Abstract

During the Ebola outbreak that hit Guinea in 2014, most of the people employed at the Wonkifong Ebola treatment unit were from Africa or Cuba. Despite the recruitment of black personnel, the unit exposes how the humanitarian infrastructure exploited Guinean workers as if their lives were less vulnerable than those of the foreign personnel. The Africanization of aid reveals a post‐colonial segregation at the intersection of race, class, and locality. The article follows Guinean workers in the quarantine unit, as well as their enrolment in media campaigns. Their experience illuminates a triage at the core of Global Health according to which not only were local workers treated as expendable lives, but their stories were silenced. Yet how did Guinean workers inhabit this anti‐black world? The article unfolds the journey of workers during the outbreak and three years later, exploring the strategies they used to produce their own narratives through personal archives. *[humanitarian aid, humanitarian media campaigns, race, Ebola, archives]*

## Résumé

Durant l’épidémie d'Ebola qui frappa la Guinée en 2014, la majorité des soignants du centre de traitement Ebola de Wonkifong provenait d'Afrique ou de Cuba. Cependant, malgré le recrutement d'un personnel majoritairement noir, l'infrastructure humanitaire a exploité les travailleurs guinéens comment si leurs vies étaient moins vulnérables que celles du personnel étranger. Ainsi l'africanisation de l'aide met en lumière une ségrégation postcoloniale à l'intersection de la race, de la classe et de la localité. Cet article explore le parcours des travailleurs guinéens dans l'unité de quarantaine ainsi qu'au travers de leur engagement dans des campagnes médiatiques. Leur expérience dévoile un tri au cœur de la santé globale au travers duquel les travailleurs locaux ont été non seulement traités comme des vies consommables mais leur histoire a également été passée sous silence. Cependant, comment les soignants guinéens sont‐ils capables d'habiter cet univers anti‐noir ? L'article suit ces travailleurs durant l’épidémie de même que trois ans plus tard, il explore ainsi les stratégies utilisées par ces derniers pour produire leurs propres récits au travers d'archives personnelles.

“I gave him life. It is quite a lot to give. It is the opposite of nothing. And the opposite of nothing is not *something*. It is everything.”

(Kushner 2018)

## A Visit to an Old Friend

**Photo 1:**
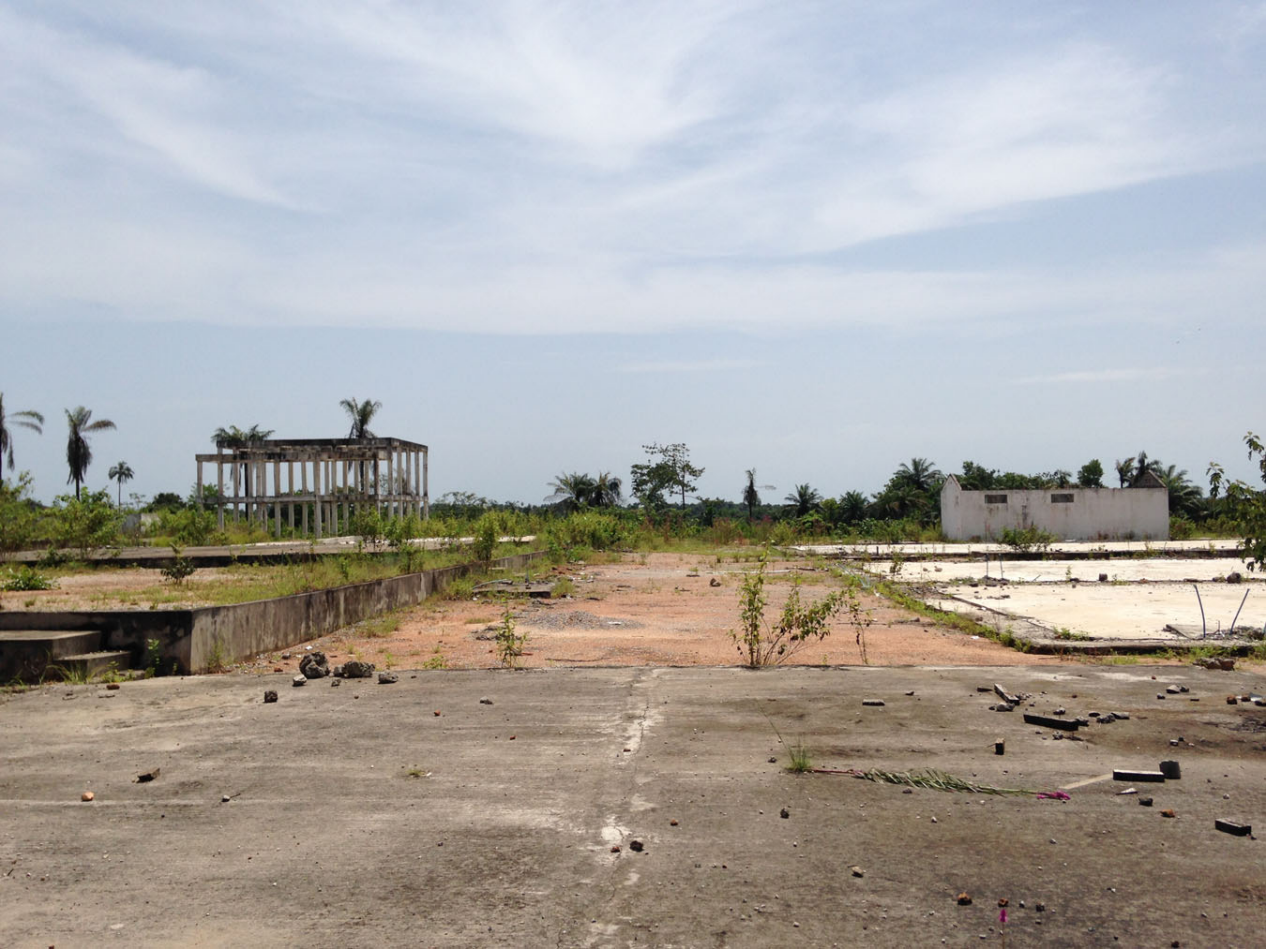
Ruins. Wonkifong Ebola Treatment Unit, 2018. [This figure appears in color in the online issue]

In 2018, I was on my way back to Guinea, where I had conducted fieldwork inside an Ebola treatment unit during the massive outbreak that hit the country in 2015 (Gomez‐Temesio 2018, 2020b; Gomez‐Temesio and Le Marcis 2017). When I had left, several buildings of the capital were covered with portraits of health workers committed to the fight against Ebola. Three years later, though, all these traces of the outbreak had disappeared. A few hours after landing, I was driving to Wonkifong in the district of Coyah, to the former Ebola unit. I had wanted to visit what was left of the unit with Sory,^1^ a former Guinean health promoter who used to work there. It was now an abandoned site, he had told me on the phone a few days earlier. Indeed, though the facility had closed only three years before, already it seemed a lifetime ago. There were no roofs or walls, just a concrete slab partly covered by the lush vegetation of Guinean countryside (see Photo 1).

**Photo 2:**
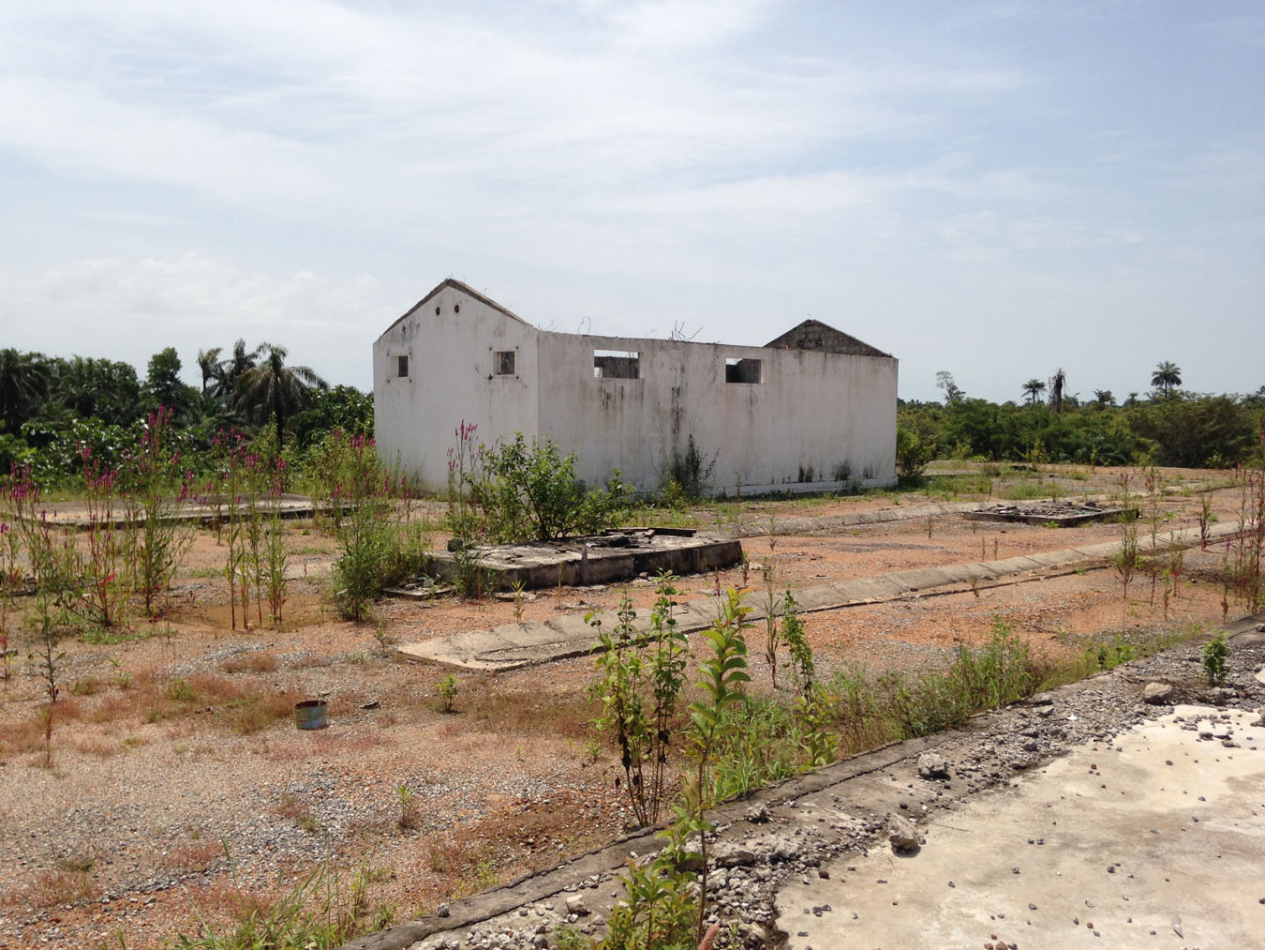
The former morgue. Wonkifong Ebola Treatment Unit, 2018. [This figure appears in color in the online issue]

Sory looked worried. He had been unemployed since the epidemic reached its end. In 2018, though the virus was no longer an active threat, it outlived that period as a kind of failed promise. Clearly, the afterlife of Ebola did not match the future Sory dreamed of when he enrolled in 2014, having decided to risk his own life to save others. He felt that this place could have represented a future for his past commitment. “I wish something had been done here. It could have been a health center. I could have worked here.” He seemed depressed. Suddenly though, he became infused with a residual energy. Jumping on a slab of the former morgue, Sory began to rehearse in mime all the routines that had flowed over that space a few years before. “When a body came, we put him there. Do you remember every time a TV crew came around here? They only got the big shots [the doctors]. But I had so much to tell! Would you film me now as you did when we worked together?” Indeed, during fieldwork, I used my phone to film the unit's daily life. I started modestly, just to keep a trace of the infrastructure. But then Sory turned out to be inclined to star in what he called “my movies.” And I began to film and take pictures of him more regularly (see Photo 2).

Wandering among the ruins, Sory remembered one film that took place during a chaotic day in 2015. Not only had a patient infected with Ebola escaped from the isolation zone, but he had been overcome by diarrhea just in front of the staff quarters. The foreign personnel—Cubans, Congolese, and a few Europeans—complained about the mess to the Guinean director. They threatened to lodge a complaint with the World Health Organization (WHO), who officially ruled the place in collaboration with the national government. The patient escaped through a door that had been damaged by an ambulance. Still, the director ordered Sory to clean up the feces. Some workers laughed while he was performing this dangerous task. He shouted at the mocking audience and asked me to start filming: “What is it that you don't understand? I am ready to sacrifice. You do know Henri Dunant? He founded the Red Cross. I live by his ideal. I have volunteered many years at the Guinean Red Cross. I don't care about doing this.”

Sory wandering among the ruins of Ebola embodies the focus of a long‐lasting ethnographic project that brought me to Guinea many times between 2015 and 2018. Following Sory's experience during the outbreak—as well as his own souvenirs of his journey—this article explores anti‐blackness in humanitarian aid through the lens of risk and testimony. Not only were Guinean workers treated as expendable lives, but their stories were also neglected—censored or confined to personal archives. While the humanitarian “localization agenda” (Duclos et al. 2019) and more broadly the “Africanization of aid” (Issoufou 2018; Redfield 2012) increasingly push toward hiring local personnel instead of Western expatriates in relief projects, the risks these workers are exposed to dramatically increase without their benefiting from the same incentives as white/Western employees. But as Sharpe warns us, the “non‐ending subjection” of blackness also relates to the treatment of black experiences in the public sphere (Sharpe 2016). In 2014, *Time* magazine elected the “Ebola fighters” personality of the year.^2^ It seemed then that people who were ready to sacrifice themselves were enthusiastically celebrated. But is this true for everyone? Here, I address the post‐colonial reconfiguration of race in humanitarian aid in two different segments of humanitarian infrastructure: within the mundane details of daily aid relief and within the organization of media campaigns.

**Photo 3:**
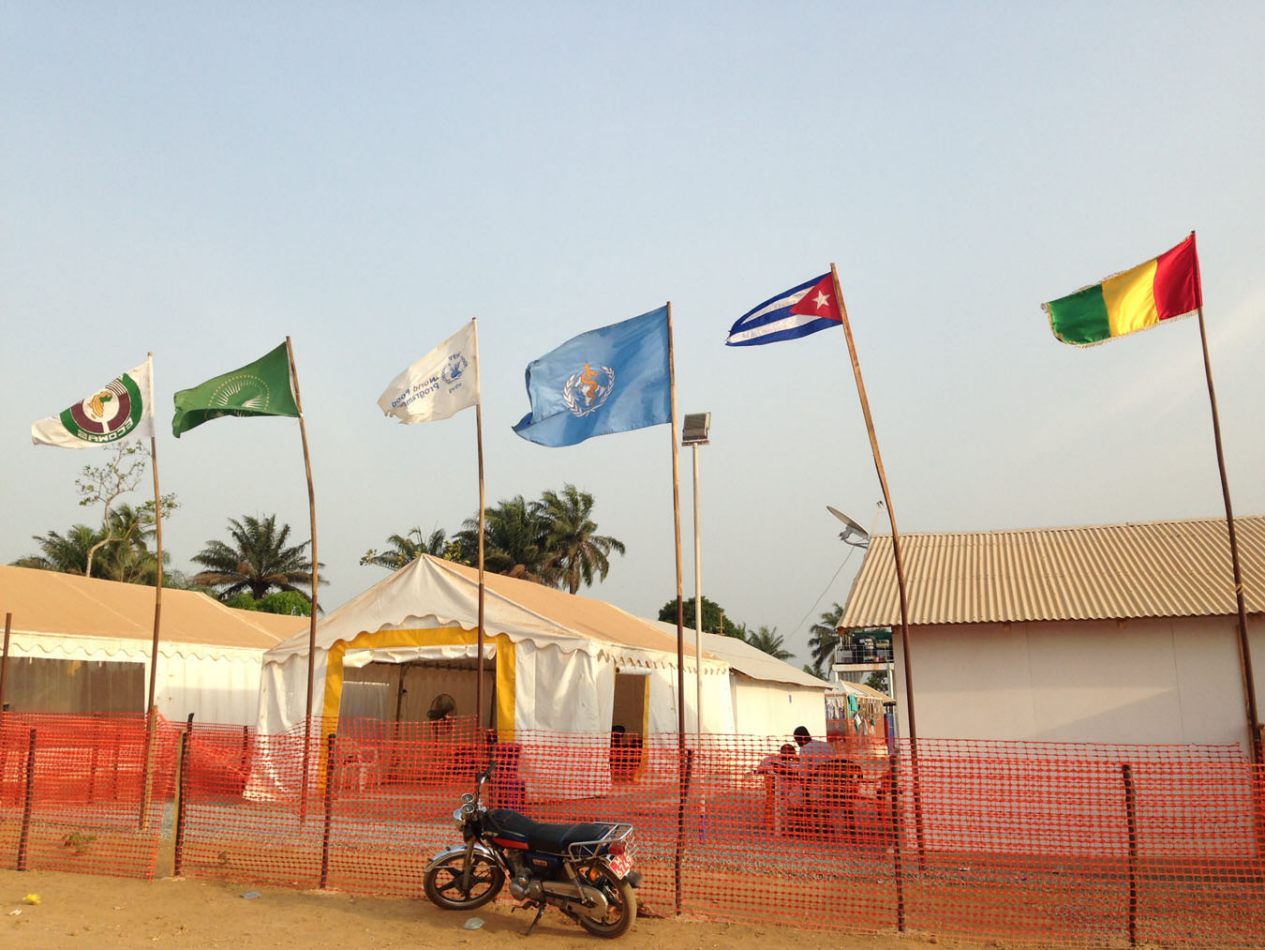
South/South collaboration. Wonkifong Ebola Treatment Unit, 2015. [This figure appears in color in the online issue]

Humanitarianism contains within it the most banal and yet unutterable truth. Not all lives have the same value; some can be risked, while others can only be saved. While the literature on humanitarian aid mainly explores the differential value of life between aid workers and aid beneficiaries (Fassin 2007, 2009, 2018; Packard 2016; Redfield 2013), little has been written on local aid workers and even less on the role of race (Benton 2016a, 2016b). This article, among others (Hirsch 2019, 2021; Issoufou 2018; Kingori and McGowan 2016), addresses this gap, shedding light on the enrolment of local workers in Guinea during the Ebola outbreak, the increased risk to which they were exposed, and their limited access to humanitarian media campaigns (see Photo 3).

In the Wonkifong Ebola treatment unit, where I conducted fieldwork in 2015,^3^ tasks inside the unit were segregated according to race and locality. The unit had been built by the World Food Program as part of the UN commitment to fighting the outbreak. At its inauguration in December 2014, it was the largest isolation infrastructure in the country and could accommodate up to 100 patients. The Wonkifong unit grew out of a South/South collaboration under the auspices of the WHO. Inside the unit, Guinean health workers collaborated with health teams from the Democratic Republic of the Congo (DRC), deployed by the Economic Community of West African States and the African Union, as well as a Cuban medical brigade hired by the WHO. A French nurse and a Haitian logistics specialist were hired to control functional administration and a European lab processed all the testing. The foreign staff thus consisted mainly of doctors, nurses, and epidemics experts. Guinean workers, meanwhile, were mostly employed as *hygiénistes*, or aides. They were assigned the most dangerous tasks, such as disinfecting contaminated objects or handling corpses. Most of these workers had no proper medical or humanitarian training. They were mainly male and female youths from the region, unemployed before the outbreak.

That chaotic day in 2015, once he had finished cleaning up after the escapee, Sory told me: “You know it's true, I always wanted to study medicine. As an orphan, I had to quit school. But my dream was to save lives.” Sory was bitter. Earlier that week, journalists had come from Cuba to film a news story. As the journalists mainly filmed their own *compatriotas*, Guinean workers only appeared in the background and were asked questions such as “How do you feel about Cuban citizens who have come to Africa to risk their lives?” Media campaigns took place regularly in the unit. They were mostly conducted by UN agencies or else were initiatives taken by one of the medical contingents.

**Photo 4:**
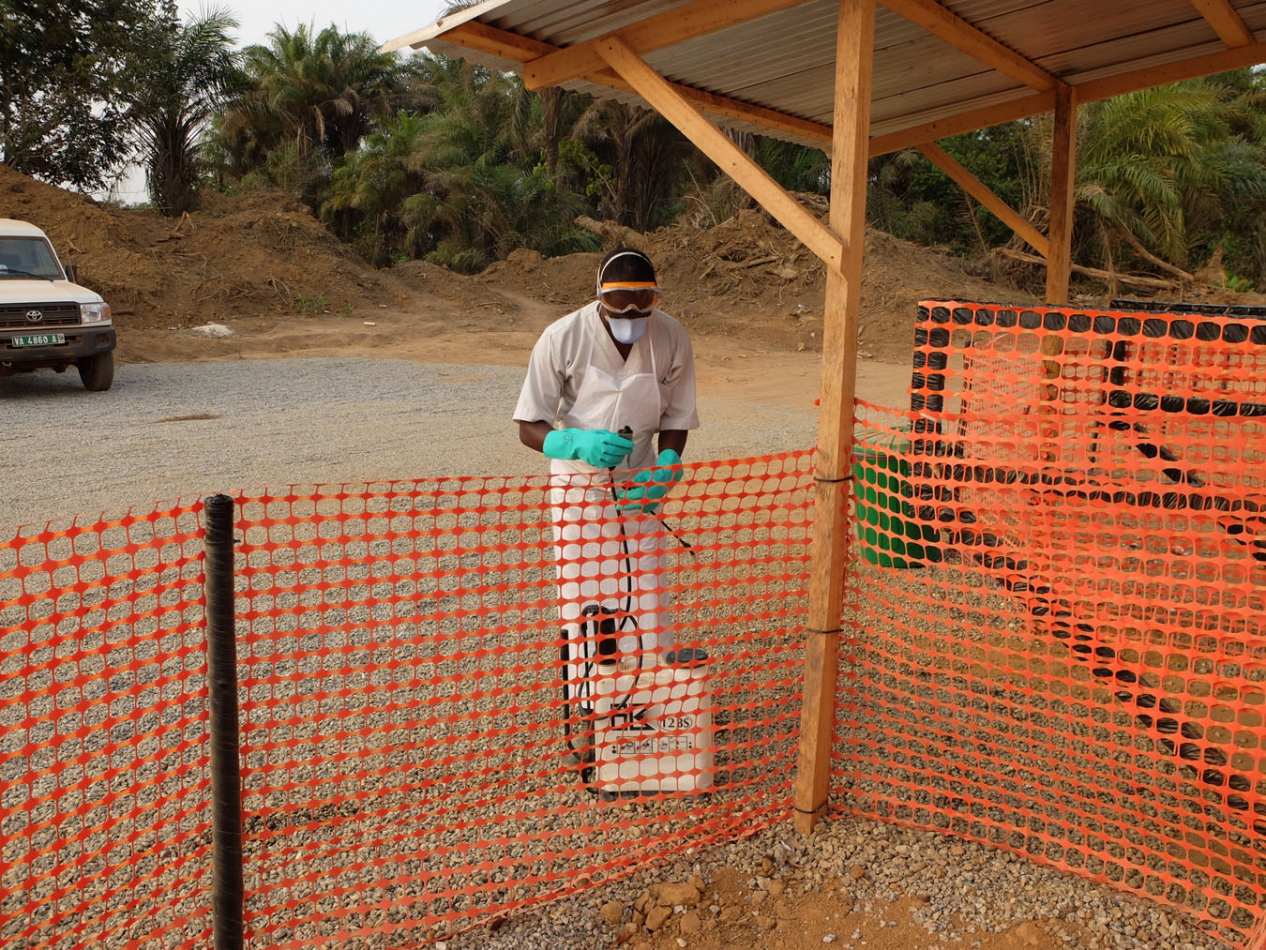
Sory cleaning after the escapee. Wonkifong Ebola Treatment Unit, 2015. [This figure appears in color in the online issue]

Advocating for the victims of the Global South is key to the rise of what Fassin identifies as the second age of humanitarianism, introducing an important distinction into the public arena between “those who are *subjects* (the witnesses who testify to the misfortunes of the world) and those who can exist only as *objects* (the unfortunate whose suffering is testified to in front of the world)” (Fassin 2007: 517). Nevertheless, when Fassin identifies two types of humanity, he leaves out how race shapes the ability to testify at the heart of the humanitarian apparatus: among its own employees. Indeed, during the outbreak, humanitarian media campaigns had one thing in common. They hardly ever gave voice to local aid workers. Nevertheless, my argument here does not steer toward a final account of exclusion. The archive, writes Garcia, may “open up the possibility of new historical narratives and modes of subjectivity” (2016: 575). Exploring daily life in the unit, I investigated how Sory and his coworkers were able to tell their own stories through alternative mediums and in intimate circles, and notably through what I would describe as private archives: recorded interviews, pictures and private movies, together with material memories from the unit that represented for them proofs of their involvement (see Photo 4).

An archive, continues Garcia, is “like a place for things you want to save. … Not like the self‐storage units people rent … but more like a photo album” (2016: 574). In Wonkifong, I progressively focused an important part of my fieldwork around Sory, and, somehow, we started archiving together. We composed a photo album of his journey during and after the outbreak. Sory liked to share his feelings, and something drew me to him over the other workers. Our interactions were influenced by economic and racial inequalities. I was a white European scholar, and he was a Guinean man engaged in a constant struggle to make a living. Yet as we got to know each other, we shared confidences. I was born from parents who had fled a dictatorship. As the child of a man who experienced extreme violence and until his death was denied political recognition, I always felt drawn to write about others whose stories had been silenced. Sory had lost his father early in life, and this loss could be narrated as the first injustice that opened the door for others: lack of education, financial struggle, the absence of recognition. We both recognized the misfortunes of our fathers as decisive, and in this common understanding we met. Sory always enjoyed the idea that, as well as writing about the work of Wonkifong, I was writing about *him*. While scientific publications were my own project as a scholar, I came to realize that we also shared a more participative project: *archiving*. And that through this archival project, Sory was also able to produce his own narrative.

About private archives, Garcia notes that they constituted “texts that embody the key meanings of loss and survival” in her field of study (2016: 575). Accordingly, when doing fieldwork, I focused on how local workers fought silenced stories. These narratives eventually came to be at the core of my ethnographic journey. They shed light on how Guinean workers resisted the *de facto* relegation of their role by the media, and thus produced their own stories at the heart of an anti‐black world. My ethnographic method in the unit, based principally on participant observation and informal discussions with local workers, at the same time produced data and created a space where they could share their thoughts and be recognized by a much wider audience for the journey they had experienced. Keeping a record of our exchanges, this article then established a dialog between the ethnography of a space, its rules and daily life, and the kind of archives that ethnographer and informants might build together, as well as the ones the latter constitute on their own. This project translates here into three different sections. In the first one, I explore the postcolonial reconfiguration of race in humanitarian aid, or how anti‐blackness dictates segregated exposure to risk even between persons coming mainly from African and Caribbean regions. In the following section, I explore a media campaign that took place during the outbreak and discuss how local stories were silenced. I then show how I helped curate the private archives of local workers’ journeys through my ethnographic involvement in the unit. Finally, a leap in time shows how, in 2018, a secret archive kept by Sory came to embody his own sense of care and sacrifice and helped him live in the present despite the social consequences of the outbreak.

## The Enduring Exposure of Black Bodies in a Humanitarian Infrastructure

Humanitarianism, warns Fassin, unfolds a complex ontology of inequality that differentiates in a hierarchical manner the values of human lives (Fassin 2007). In making this argument, he builds on Hannah Arendt's theory of political recognition. Those in the category of *bios*, a politically recognized life—for Fassin, humanitarian workers and, for Arendt, Roman and Greek masters—are contrasted to *zoë*, a biological life common to animals and humans—the beneficiaries of aid for Fassin, the slaves of antiquity for Arendt (Arendt 1998 [1958]; Fassin 2009). While Fassin focuses on the humanitarian dichotomy, Arendt draws more broadly on life's daily tasks in antiquity. As she explains, the free man *works* with his hands, and the slave *labors* with his body, thereby freeing the masters from the most painful work (1998 [1958], emphasis added). Hence, the segregation of tasks, or the difference between work and labor, is key to understanding who is considered human (therefore having a political life), and whose life is excluded from the realm of humanity. While neither Arendt nor Fassin acknowledged race as crucial to the value given to different groups of people, more recent work on the entanglement of race and medicine sheds light on the enduring exposure of black lives to greater risks, whether in colonial and slave hospitals or under care conditions in contemporary and humanitarian medicine (Gomez‐Temesio 2018, 2020a; Gomez‐Temesio and Le Marcis 2021). The concept of a black medical superbody, as proposed by Owens, points to an enduring entanglement between race, non‐humanity, and labor since the premises of modern medicine were formulated (Owens 2017). While white doctors dismissed black bodies as completely other, they still exploited them as guinea pigs because they thought they could endure more pain than white bodies, pictured as more vulnerable and constituting lives of greater value. More recently, writing on COVID‐19, Oyarzun (2020) explored the intersection of race and class among civil servants “volunteered” by the state on the frontline of the COVID‐19 pandemic. From colonial to contemporary times, race dictates a segregation of tasks in medical contexts through which black labor is equated with productivity and invulnerability.

**Photo 5:**
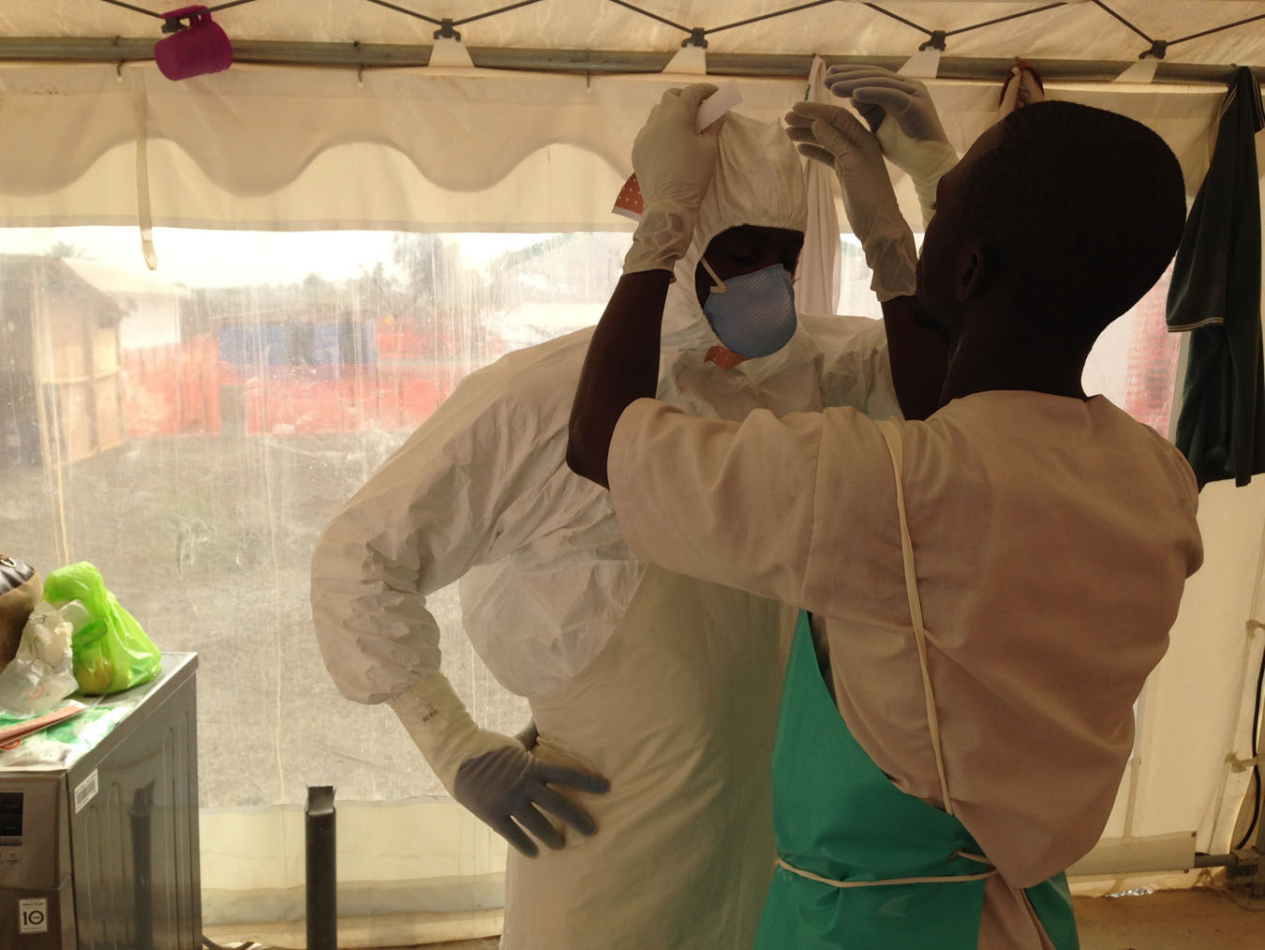
Dressing for the quarantine zone. Wonkifong Ebola Treatment Unit, 2015. [This figure appears in color in the online issue]

In Wonkifong, the segregation of tasks between foreign and local personnel is thus emblematic of an inequality that was shaped not only in two different humanities—one biological, the other political—but also in terms of race and class. In the transnational space that the unit constituted, Guinean caregivers were assigned the more exposed tasks. They cleaned patients and furniture with chlorinated water. They incinerated patients’ belongings. They managed corpses. They handwashed all personnel uniforms with chlorinated water. Yet local workers were much more exposed to contaminating fluids (see Photo 5).

Behind the division of labor were important discrepancies in terms of salary. Foreign workers were paid much more than Guinean workers while also enjoying benefits such as daily allowances and professional opportunities at the end of the outbreak. Though Cuban and Congolese earned more than Guineans, their salaries were still ridiculously low compared to those of the Western staff employed at the WHO headquarters in Conakry. Wonkifong was hence the biggest unit of the country while being at the same time the cheapest, employing almost exclusively African and Caribbean low‐wage workers as well as Guinean personnel working for even less. Basing aid relief on cheap local labor allowed the deployment of a massive Ebola response in the region (Kingori and McGowan 2016) to protect Western countries from the deadly virus. Consequently, Wonkifong's division of labor enlightens the fact that the “politics of life” (Fassin 2007) that granted less value to the lives of aid beneficiaries also applies to African aid workers (Benton 2016a, 2016b). Therefore, this inequality does not operate vaguely on different groups of humans but, instead, on different groups of racialized and working poor people.

Yet Wonkifong constituted a peculiar anti‐black world in that, unlike most of the Ebola infrastructures around the Manor River region (Hirsch 2019, 2021), there were almost no white or Western personnel working there. Its employees were mainly black people coming from Africa and Cuba.^4^ Wonkifong thus challenges post‐colonial imaginaries around a binary division between white and black people (Oyarzun 2020), as the segregation of work operated not in terms of skin color so much as in degrees of closeness of black people to white power. Accordingly, the endurance of racial hierarchies also translated through projections of whiteness.

Ezequiel was a Congolese logistician. Some said behind his back that he was close to the DRC secret services and had been dispatched to Guinea to keep an eye on the situation there for his government. True or not, Ezequiel was obeyed by most of the staff. He never went into the quarantine zone but was strategically in charge of the unit's warehouse. His authority was translated into the nickname he was given: “*le Blanc*,” the white man. Once, Ezequiel got sick and vomited in the middle of the unit, greatly disturbing his coworkers. A Guinean worker remembered, “When we saw le Blanc, it was terrible: If he got Ebola, what about the rest of us? If le Blanc can get sick, then we are all really screwed.” Ezequiel's whiteness projected authority but also privileges. Ezequiel, as a figure related to whiteness, was not supposed to die of Ebola, unlike his other African colleagues. This anecdote underlines the shared perception of the Guinean staff that privileges were restricted to white people or those close to white power. Ezequiel's supposed proximity to the DRC secret services also sheds light on post‐colonial imaginaries that equate corrupted African elites with former colons and contemporary white power. Consequently, Wonkifong provided a perfect setting in which to investigate the post‐colonial reconfiguration of race as an integral part of the Africanization of aid.

The psycho–social team of which Sory was part dealt with all the paramedical work of death and life: admissions, family visits, and authorizations to bury. Local workers were thus more exposed to local resentment. One morning, a patient died. Sory called the relatives of the deceased, whose contact details were in the unit's patient register. This book was the most valued item of the unit. Sory's team managed it even though doctors came regularly to check some information, as it was the only place where all the patients’ information was gathered. The family came to the unit. In accordance with the biosecurity protocols, the corpse was shown to them in a bag through a fence.^5^ When the two young aides lifted the body of the deceased, one of them feared his gloves would tear under the pressure. He panicked and dropped the body. The corpse fell on the ground. The relatives, deeply shocked, started to scream. One told the aides, “Lucky you, we have no guns. Otherwise, we would have shot you.”

This was hardly uncommon. A few weeks before this incident, a team of workers were assaulted by a mob. Therefore, security in the unit and at the hotels where the foreign workers were staying was increased. Guinean workers, however, enjoyed no such “institutional immunity” (Fassin 2007: 515). Sory said he had to stop going to the market after people threatened him. Indeed, most workers endured social ostracism. Fatie worked alongside Sory in the psycho–social team. She was an agro–economist who had never managed to find work in her field of study. When she started working in Wonkifong, she was disowned by her fiancé, with whom she had a child. She said, “I don't know if we can go back together. His family opposes it. They said I kill people here.” Fatie had to leave her infant with her family while she shared a house with colleagues on the outskirts of Wonkifong.

Labor and work also implied a segregation of space between different groups. Wonkifong had two main spaces: the quarantine zone, where infected patients were isolated; and the rest of the unit, composed of medical and administrative quarters. There was a cafeteria where a local cook prepared meals for a fee. This place was open to everyone but was in fact only used by all the Guineans and some Congolese workers who had no other option. Cubans went back to their hotel, where they enjoyed their own menu, as did some of the Congolese. Europeans at the lab enjoyed meals delivered daily—initially, it seemed, for the patients—by the World Food Program. The cafeteria was hence serving local food for Africans. Hardly anyone was happy with the place. The food was described as too expensive and not sufficient. As Sory put it one day, it was as if “the cook split one corned beef can between 50 people.” Plates and spoons were also in short supply, and workers had to share. Collective diarrhea cases were reported. African workers were therefore much more exposed to contamination if Ebola was to spread among the staff.

While local workers took disproportionate risks in comparison to their foreign colleagues, they were not guaranteed the same conditions if they suffered contamination from Ebola. Indeed, while repatriation was a guarantee for foreign workers, Guinean personnel had to be treated in a unit within their country. If the Ebola mortality rate was around 70% in the Mano River countries, it fell to under 20% for repatriated humanitarian personnel (Farmer 2015). The life expectancy of an aid worker could not be determined only on biological grounds (Canguilhem 1966); it also related to work segregation. The journey of local workers thus revealed the dialectics between race and capitalism from colonial times to the era of Global Health, as while black subjects were segregated in every aspect of daily life, their access to low‐paying employment still had to be guaranteed.

## Walls of Hope, Voices of Oblivion

Since the Biafran war, advocacy has become one of the key elements of humanitarian action in the Global South. Bearing witness, as noble as it may seem to be, still differentiates two sorts of life in the public space: those who can tell their own stories, and those whose stories can only be told by humanitarian institutions (Fassin 2007). During the outbreak, many campaigns publicized the humanitarian commitment and the need to “fight the virus” around the globe. But what lies beneath such mobilizations? In this section, I explore a visual and digital campaign that took place during the outbreak to shed light on how race segregation was embodied in humanitarian media strategies. While black heroes appeared in these campaigns, the voices of Guineans were systematically excluded from the limelight.

**Photo 6:**
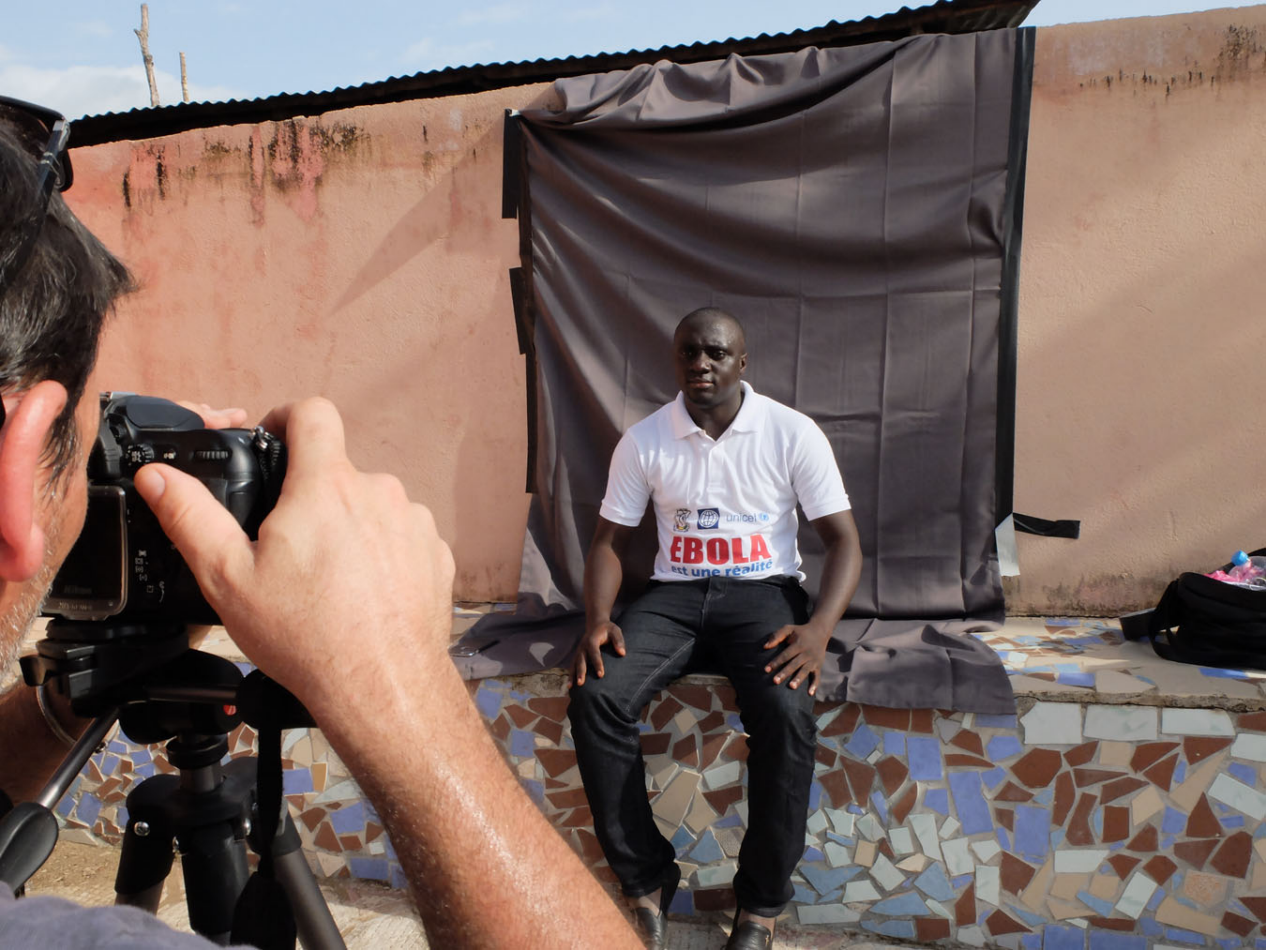
Pictures and testimonies. Forécariah préfecture, 2015. [This figure appears in color in the online issue]

“Tribute to Ebola Health Workers”^6^ was a campaign launched by the United Nations Development Program (UNDP) in 2015, during which portraits of individuals committed to the Ebola response were posted on the walls of several buildings in Conakry. The campaign was accompanied by the collection of workers’ testimonies, archived on a website. I participated in the project by chance when I was asked by an acquaintance at UNDP to gather the testimonies. He thought that as an anthropologist with experience of working in an Ebola unit it would be easier for me to make contact with humanitarian workers (see Photo 6).

We interviewed foreign and local personnel in several of the Ebola units in the country as well as at the WHO headquarters. The atmosphere of the recollection changed dramatically depending on the interviewees. Expatriate workers, used to such media campaigns, mostly had ready‐to‐go slogans like “Together we can!” In contrast, when we interviewed Guinean workers, they were more eager to share an actual experience. One young man even came back later to give me a letter in which he had recorded his own participation since the beginning of the outbreak. Guinean workers spoke about the patients they had lost, but also about their own losses. Some were driven out of the house they lived in, while others were left by their spouses. They all emphasized the difficult tension between their role during the outbreak and their future return to “real life.” The most recurrent themes were messages like “Now that our parents consider us as murderers, who is going to care for us at the end of the outbreak?” or “We want jobs after the epidemic, we don't want to go back to being unemployed.” Many mentioned initial studies and prior employment—historian, lawyer, engineer in micro‐finance, or salesman for a phone company. Several mentioned having been employed by mining companies. As the outbreak brought mining activities to a standstill, they were suddenly left without income. Hence, Guinean testimonies were not restricted to their experience as Ebola fighters, but also dealt with employment in their country as a disappearing phenomenon. As a Guinean aide confided to me:

The worst problem in Guinea, it is not Ebola, it's poverty. We barely survive in this country, and then when Ebola came, people had no money to avoid the virus. Guineans, they might accept that the only solution is to die. The government did not move a finger for us. Did you notice? The best routes in this country are for the miners. And our hospital in Conakry? This is worse than Mozambique during the war.

The recollection process led sometimes to tensions and demonstrated how for expatriates the tribute was immediately recognized as a communication strategy aimed at “showing success” and keeping the money flowing to projects, while local workers tried to gain political recognition. In the Forécariah Ebola treatment unit, managed by the French Red Cross, the *chef de mission* interrupted the recording, infuriated by the fact that the local aides spent so much time talking. He had presented his own testimony in two minutes and was unable to contain his fury: “You all lazy, back to work or I will fire you!” The recollection was brought to a close. Guineans could not risk losing their jobs. For every story that sees the light of day, Jackson tells us, several others remain untold, censored, or even suppressed (Jackson 2002). The testimonies chosen to be featured on the project website only spoke about heroism, with slogans such as “The fight goes on!” The humanitarian apparatus has the power to constitute truth in the name of others, and here local workers were denied a public appearance as the subject of their own story. The stories displayed in the digital archives were only the fragments fitting the message UNDP wanted to communicate.

In his “Pro Patria Mori,” Kantorowicz reminds us that the sacrifice for the homeland always appeals for a counter gift (Kantorowicz 1951)—the political recognition of the value of the sacrificial individual. With the concept of “politics of life,” Fassin points out the same link between political existence and sacrifice. Only Western saviors can give their lives for a noble cause—local victims of wars and epidemics are only recognized as biological losses in the overall statistics (Fassin 2007). But what the backstage of a campaign such as UNDP's reveals once again is that racial fault lines segregated the treatment of local workers in daily tasks as in their access to media campaigns. This deprivation of political recognition was linked to the fact that Guinean workers were seen through the lens of locality and economic opportunity. As a foreign doctor told me once, “These [pointing to patients] are their people. If Guinean workers are not the first to care for them, who will? Plus, they are being paid to do so, aren't they?” Different variations along *their country, their parents, their job* were recurrent in the daily chit chat. Therefore, local workers were described as people benefiting from an economic opportunity more than demonstrating a will to sacrifice. If this interpretation was not necessarily wrong—many Guinean workers found themselves without a job because of the epidemic and thus needed a new means of survival—it still excluded them from the “counter gift of sacrifice”: political recognition (Hubert and Mauss 1925; Wool 2015). Their portraits appeared in the campaign, yet voices were silenced when the tale they told was embarrassing for humanitarian organizations.

## “We Saved Lives”: The Stories I Was Told

Wonkifong was an extraordinary place within the humanitarian landscape of the Manor River region: It offered the unique experience of running an Ebola unit with people who came almost exclusively from the Global South. But the place was excluded from the UNDP tribute for trivial concerns. The recollection was performed and filmed during the rainy season. The road was in poor condition and the producer decided to stay in Conakry so that we could focus on other structures—such as an Ebola unit run by the French army and the WHO headquarters.

When I eventually returned, I found the local staff at Wonkifong discussing a strike. The outbreak had faded out in the region, and the unit was slowly closing down. Most of the local workers had been notified of contract termination. They were offered no prospect of future work. Sory hardly slept at night and feared for his own life. Fatie was scared. She had nowhere to live, as her whole family rejected her. Ebola fighters from elsewhere gained a public tribute as they fitted the archetype of humanitarian heroes. Guinean people and their mundane difficulties, such as unemployment and social ostracism, were left in the dark.

But even though they were denied a space within the big tent of humanitarian aid to share their experience, memorialization was a common concern and inner need for local workers. Most of them regularly took pictures while working. This material was eagerly shared on social media during and after the epidemic. At the end of the tribute, I had spent six months in the unit. I rarely recorded our conversations, as I preferred them to remain informal. But after the closure of UNDP's project, I thought recording might be an interesting approach. I asked the workers what they wanted to tell, to remember. Most of them spent considerable time speaking about the patients they had lost, but they also wished to remember those they had helped to survive. Sory recollected how he had once experienced a conflict with Cuban doctors. It was about a two‐year‐old boy who had been quarantined alone with no family members present. Sory opposed the placement of the child in the women's tent. He remembered the Cubans gossiping about him: “Who wants to put a kid with unknown males?” But Sory had his reasons: “All the women in the tent were nearing death. ‘‘In the men's tent, on the contrary, there were quite a few men who were already recovering—they could feed the baby”. Sory changed the location of the infant at night, when the foreign doctors went home. The next morning, one Cuban almost beat him. Still, the boy remained with the men. He was fed by them—and ultimately he survived.

The memory Sory decided to share was highly relevant. According to foreign staff, local aid workers did not abide by all the security protocols or respect collective representations on how to handle sick people. The way the Cuban brigade welcomed me at the unit was emblematic of this. Their chief, probably assuming that as anthropologist I was there to deal with the “natives,” told me: “*Guineanos*, they don't really know how to care for their own. It would be great if you could keep an eye on what's going on here.” For most foreign workers, the locality of Guinean employees corresponded to a lack of education. Regarding Cuban workers, it mirrored the fact that Cuba has trained a substantial part of Guinean medical personnel over the years (Kirk and Erisman 2009). And because Ebola was first discovered in The DRC (Piot 2012), Guineans were seen as those who let the virus spread into the region, while the Congolese had always managed to stop an Ebola outbreak before it threatened another country.^7^ Therefore, being local was translated into “having to learn something” from foreigners, as well as sharing the blame for the outbreak with the patients quarantined in the unit.

Yet, local workers often found creative ways to care for those in their charge. One worker told me how she used to trick patients to build trust. As she was hidden under her protective equipment, patients could identify her only by her voice, speaking in the different local languages. She usually checked their details in the patients’ register and then made up a character identity that she could assume in conversation with the patient. Once a young man refused to eat for an entire day upon his arrival. She explained:

He came from a village I knew. I told him, “Hey, do you recognize me? It's me who sells you the best plantain at the stall close to the gas station.” It worked! I could see he was relieved. I explained to him, “There is nothing to fear here, just eat and take the drugs, you will get better.” She laughed at that memory but while we went on with the recording, she got sad. “There was this little boy. He didn't want to eat. I said to him, ‘‘Don't you recognize me? I am your aunt, I know your mommy very well.”^8^ His eyes became bigger. “My aunt?” “Yes, I came to see if you are eating to feel better. Please, drink some juice if you want to go back to school.” He said, “*Ma tante*, but I don't go to school.” I replied, “If you take your juice, I will send you to school.” He seemed so happy. “Auntie, is it true? You are going to send me to school?” He drank but vomited immediately after. I could see he was not going to make it. I started crying. A doctor summoned me. “You can't cry in front of the patients.” The day after that, I went to the morgue to check on the corpses. He was there, my little boy. Till this day I remember him.

Fatie also took great pride in the fact that she helped people change their minds about the quarantine unit around her:

My own *maman*, she disowned me. She said I will bring the virus with me and kill her. But then once I asked a colleague to film while I was dressing in the protective equipment. I shared it with my mom, friends, everybody! People understood we were safe, and that we were helping people who got sick.

Guinean workers spoke most about how they managed to make a difference, and about the patients they had lost and were unable to forget. These memories highlighted the fact that if local workers could not share their own stories on humanitarian media campaigns, they could—and knew how to—work. They saved lives—even though their foreign colleagues and the international media campaigns did not acknowledge them.

## Of Archives and Love: Three Years Later

**Photo 7:**
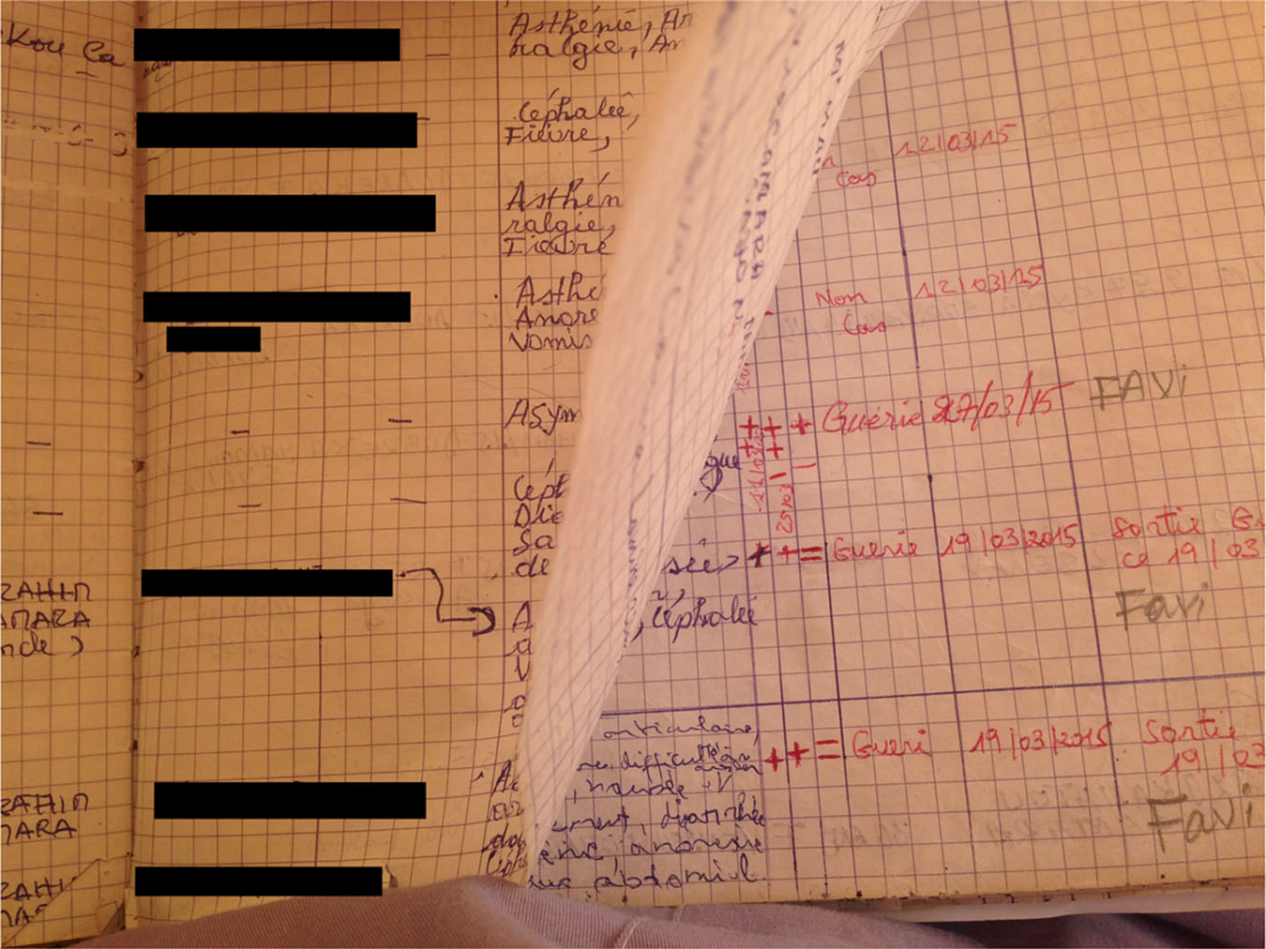
The forgotten archive. Coyah préfecture, 2018. [This figure appears in color in the online issue]

We are back in 2018. Wandering among Wonkifong's ruins, Sory and I remembered those who worked there. “Have you seen pictures of Claude on Facebook?” he asked. “Apparently, he is on some humanitarian mission in Asia.” Until recently, Sory had kept in touch with his former foreign colleagues. But then his computer broke. He had no money to fix it and then didn't get news from Claude and the others. He kept in touch with the Guineans, though. He told me that Fatie had passed away a few months before. Sory explained. “She had another child. I am not really sure how exactly, but she died afterwards.” I remembered her being scared at the closure of the unit. Sory continued: “She got sick right after the baby was born. She got anaemia and, I guess, had no money for the drugs.” Fatie was a young mother who had been willing to sacrifice her existence to fight the virus, and then succumbed while birthing another life. She gave the gift of life three times and then died because she was unemployed. She was 32 years old (see Photo 7).

When talking about Fatie, her enthusiastic voice on the recordings came to me and I remembered her careful writing in the patients’ handbook. It seemed to me that this book constituted the most important archive of Wonkifong. I wondered suddenly what had become of it. Sory smiled. “I kept it. No one asked for the register when the unit closed. So, I just left with it.” Sory did not get to tell his story, but he became the guardian of the stories untold. He revealed, “Nobody knows I kept it. Sometimes I go through it. I remember the people.” A self‐made curator with no gallery or museum of his own, Sory nevertheless protected his story within the intimacy of his home.

The fact that no one cared to retrieve this register underlined the difference in treatment accorded to what come to be considered as data and material that has the potential to become an archive. Data were produced by the different medical contingents during the outbreak. Data translated patients’ experience into numbers and focused on such things as survival rate, immunity, and so on. The patients’ register contained information that was considered as data and thus extracted in digital files, but it also remained a material trace of remembrance as it listed every patient with names, home, family contacts, and most of all, anecdotes of their stay inside the unit. The data, extracted from the medical register, were shipped out of the country at the end of the outbreak when foreign teams flew back to their countries.^9^ The register was almost forgotten in an abandoned site and eventually rescued by Sory. It then became a personal archive for him. Sory said he feared people from clinical trials might get hold of his register. “Today, everybody does money out of the survivors. With this book, I am the only one who is able to locate all of them.” Keeping the book, Sory eventually fulfilled his own dream of caring for others, during and after the outbreak. As recounted by Garcia, being the curator of her informant's prison correspondence, the register became for Sory a material remain that condensed affective and social relations (Garcia 2016: 574). An impromptu archivist of the history of the outbreak in his country, Sory continued to follow his calling in 2018, as in 2015.

While we were talking about the patients’ register, Sory suddenly said: “You are in the book.” I knew this to be the case, as back in 2015 I had spent hours filling it in, but his smile was enigmatic. I waited for what seemed to be another secret he wanted to share with me. He gave me a photograph of a bride from his pocket. “I could not tell you before I saw you. I married this woman in 2016. Do you recognize her? She is a survivor. A few days after marrying her, I looked at the register, just wanting to see her details, out of curiosity. And I saw your handwriting, you're the one who signed her certificate of departure.” Since 2015, my encounters with Sory have always been structured by unequal relationships. I was born white in a Western country, my access to education drove me to work in a profession that brought me great joy and fulfilment. But when he disclosed this hidden archive, it appeared that my hand had helped shape the path of his Ebola afterlife, in which he found some sort of social recovery. Both our journeys colluded here. As children, he dreamed about becoming a doctor and helping others, while I longed to tell forgotten stories about loss and silence. In this we eventually met.

Sory told me the story of his bride. “She was desperate, not only because of Ebola. Her husband had died. I wanted her to find the strength to survive. I told her, if you do it, if you get out of this place, I will marry you when all this is over.” And so he did. By marrying a former patient, Sory gained a precious sense of recognition at a time when family and neighbors had turned their backs on him. “People still threaten me. But my wife, she is grateful.”

“Recognition appears beyond its inscription in language,” notes Le Marcis (2012: 488), “it is embodied in acts of care.” Sory said to me about his wife, “People treat Ebola survivors as waste. I wanted to show them, they are human beings.” Sory and his wife had no children of their own. He confessed that Ebola probably made her infertile, as had happened with many other former female patients. Nonetheless, he had no regrets. Happy was maybe not a term that would describe him, but he had a meaningful life, consistent with his previous commitment: “No matter what happens to me now, when I am at home, I feel good about what I did.” Later, when his wife came home, he quickly whispered to me, “Don't tell her I have no job.” He didn't want her to be worried. Sory was not recognized for the role he played during the epidemic, but his marriage somehow helped him be the hero in his own history. Looking at Sory's wife that day, nevertheless, I could not help but ask myself: Who was going to tell her story? As a widow who survived Ebola, it seemed the unique path available to her was to marry again with her former savior. Thus, recovery after the epidemic was shaped through dramatic gender inequality.

## Epilogue: Not the Only Plot

Greater vigilance is required, Erikson and Wendland warn, “to undo the multiple ways that Global Health depends on steep inequalities for its very existence” (Erikson and Wendland 2014: 3). The Wonkifong unit, the only “South” infrastructure in Guinea during the outbreak, laid bare old and new forms of discrimination that bring to light a robust continuum between colonial medicine and humanitarian aid. About the involvement of black volunteers during the COVID‐19 pandemic, Oyarzun writes that in the United States the most precarious people, those without income and health insurance, literally “threw themselves at the “opportunity” to work on the frontlines of the response against a deadly virus”—while if they got infected the State would not protect them (Oyarzun 2020: 583). Ebola—like the COVID‐19 pandemic—shed light on the many ways in which black bodies are exploited at the intersection of race, class, and gender (Gomez‐Temesio 2020a).

Wonkifong's experience shows us how both segregation of work and the organization of humanitarian media campaigns underline a failure of subjectivity regarding local aid workers. When writing about my informants’ experiences during the outbreak, how was I to avoid repeating the “oppressive gaze” (Hartman 1997) in scientific writing? My ethnographic project thus focused not only on the unit's organization but on local workers’ imaginaries, regrets, and emotions—and on how they built their own archives. “Slavery was so unprecedented,” warns Morrison,

that you can let slavery be the story, the plot. … And then you do the worst thing, which is … the center of it becomes the institution and not the people. So, if you focus on the characters and their interior life, it's like putting the authority back into the hands of the slaves, rather than the slave owner. (Morrison 2020: 25)

Accordingly, in this piece, I did not stop at “describing” the segregated treatment of local workers but underlined how people inhabit these cramped spaces and how they invest themselves in “innovative resistances” (McKittrick 2013). I investigated how people reflected on their condition and tried to access their “interior life” as far as ethnography could show this: their expectations, dreams, frustrations, and disappointments—how they made sense of the present and prepared a future where life was bearable. It is in this dialog between their thoughts, our discussions, and the material and archival traces of the outbreak that I gained access to an intimate space where the racial segregation that rules humanitarian aid was not the “only plot” (Morrison 2020). As such, the journey local workers made through the epidemic underlines how people may respond to anti‐blackness by laying claim to experience on their own terms. The article hence undertook a triple triage at the nexus of humanitarian aid and medical anthropology: the role of race within humanitarian institutions; the voices of local workers in the humanitarian narrative; and the subjectivity of those whose lives we investigate in academic writing.

Back in Wonkifong during the epidemic, I used to take my notebook with me in the unit, though I wasn't always taking notes. Most of the time, I was just busy participating in the unit's life. But when there were conflicts, people started noticing that I rushed to write what was happening. And of course, most of the time, they stopped arguing when they saw me. It became a joke, as Sory summarized it one day, “when it gets hot here, Veronica always gets the pencil out of the pocket!” One day, he said proudly to his colleagues, “Veronica is writing a book. And believe me, I talk a lot in this book.” When we were about to leave the ruins in 2018, Sory asked me. “So, your book, is it already out?” I plunged into a long explanation on how writing academic books lasts forever, but, as he looked disappointed, I reassured him I had already published a few pieces in which he was “starring.” And I added, “Of course, I changed your name so people could not recognize you.” Sory was startled. “Why on earth did you do that? Then nobody will know, it was me, all the time, it was me.” Indeed, while I always knew Sory was keen to appear under his real name, until the very last moments of writing and revising this article, I hesitated. I thought I was going against his will. But I also thought he might regret, and that I owed him some *droit à l'oubli*, a right to be forgotten in the future if he ever wanted to move on from the epidemic. And I mostly feared that, in revealing him, I revealed the identity of his wife, who never manifested a will to have her story of loss and infertility shared with a wider audience. I might be wrong, or not. In this lies an ambivalent inequity that I was never able to overcome, a final limit to my archival project built with Sory where he is forced to remain the anonymous character of a story told by me.

## Notes

1 All names mentioned in this article are fictitious.

2
http://time.com/time‐person‐of‐the‐year‐ebola‐fighters/

3 I came to be involved in Wonkifong Ebola treatment unit as part of a clinical trial. As the trial neared completion, I stayed and engaged in more general fieldwork on practices of care in times of Ebola. I completed seven months of fieldwork inside the unit in 2015. Since then, I have continued doing fieldwork in Guinea on the aftermath of the outbreak.

4 The enrollment of local aid workers to perform cleaning and social tasks was recurrent in all units of the country. However, other units run by international NGOs employed Western staff to perform medical duties.

5 About safe and dignified burial protocols, see Le Marcis (2015).

6
https://stories.undp.org/les‐visages‐de‐la‐rponse‐ebola

7 This was before the Ebola epidemic that started in 2018 in the DRC.

8 Aunt or *tantie*, is a woman from the mother's generation who is not necessarily biologically related; she can be a neighbor, friend, and so on.

9 In 2019, an investigation led by journalists of *Le Monde* revealed that contaminated blood samples and biological material from Guinea, Sierra Leone, and Liberia have been taken out without respecting either ethics or legal protocols. https://www.lemonde.fr/afrique/article/2019/01/22/ebola‐l‐utilisation‐opaque‐des‐echantillons‐sanguins_5412571_3212.html?fbclid = IwAR0VaXbkKf3Csr0oaPa8AievfzaPsFHUTE3mUWL3kKqRr_d‐hlhstWlhzUk
